# Adult and neonatal models of chemogenetic heart failure caused by oxidative stress

**DOI:** 10.1172/JCI178251

**Published:** 2024-03-14

**Authors:** Fotios Spyropoulos, Apabrita Ayan Das, Markus Waldeck-Weiermair, Shambhu Yadav, Arvind K. Pandey, Ruby Guo, Taylor A. Covington, Venkata Thulabandu, Kosmas Kosmas, Benjamin Steinhorn, Mark A. Perrella, Xiaoli Liu, Helen Christou, Thomas Michel

**Affiliations:** 1 Division of Newborn Medicine, Department of Pediatrics;; 2 Division of Cardiovascular Medicine, Department of Medicine; and; 3 Division of Pulmonary and Critical Care Medicine, Brigham and Women’s Hospital, Harvard Medical School, Boston, Massachusetts, USA.

**Keywords:** Cardiology, Heart failure, Mouse models

**To the Editor:** With preterm infants often surviving into adulthood, prematurity has been identified as a risk factor for heart failure, leading to the recognition of a new disease entity: “heart failure of prematurity” (1). Preterm infants are susceptible to oxidative stress because of increased perinatal exposure and lack of antioxidant defenses (2). Oxidative stress also causes heart failure in adults and contributes to cardiovascular disease (3). Deranged oxidant signaling is a common pathway implicated in heart failure both in adults and in the perinatal period. We here report a heart failure model that permits dynamic regulation of reactive oxygen species both in adults and in the developing heart.

We utilized a chemogenetic approach exploiting a yeast d-amino acid oxidase (DAAO) that generates hydrogen peroxide (H_2_O_2_) upon provision of d-alanine (4). We previously used adeno-associated virus serotype 9 gene transfer to target DAAO expression to cardiomyocytes in vivo and showed that chronic generation of H_2_O_2_ causes heart failure (4–6). Here we generated a cardiomyocyte-specific transgenic mouse line (DAAO-TG^Car^) expressing DAAO as a fusion protein with the H_2_O_2_ biosensor HyPer allowing for simultaneous generation (DAAO) and detection (HyPer) of H_2_O_2_. We validated cardiomyocyte-specific DAAO expression and verified production of H_2_O_2_ upon addition of d-alanine (Supplemental Figure 1; supplemental material available online with this article; https://doi.org/10.1172/JCI178251DS1). Adult DAAO-TG^Car^ mice developed heart failure after in vivo treatment with d-alanine. Cardiomyocytes from DAAO-TG^Car^ mice showed higher baseline H_2_O_2_ levels after in vivo d-alanine treatment, indicating a higher intracellular oxidized state. Feeding with d-alanine increased mitochondrial superoxide (O_2_^.–^) in cardiomyocytes from adult DAAO-TG^Car^ animals, decreased mitochondrial membrane potential, decreased respiratory capacity, and increased baseline glycolysis, providing evidence of mitochondrial dysregulation as a characteristic of cardiac dysfunction (Supplemental Figure 2, A–D).

For the neonatal model, we generated heterozygous DAAO-TG^Car^ pups by crossing homozygous male DAAO-TG^Car^ sires with WT females so that all pups expressed DAAO. We induced in utero oxidative stress by providing d-alanine or l-alanine (control) in the mothers’ drinking water from embryonic day 8.5 until birth. Neonates exposed to d-alanine in utero had a decrease in cardiac function compared with controls, accompanied by decreased cardiac wall thickness and increased end-systolic volume. Neonatal cardiomyocytes isolated following in utero d-alanine showed deranged redox balance, elevated H_2_O_2_ and mitochondrial O_2_^.–^ levels, along with mitochondrial dysfunction (Figure 1). Neonates exposed to d-alanine showed increased cardiomyocyte apoptosis and proliferation (Figure 1, D–F), decreased cardiomyocyte size, and disrupted cardiomyocyte architecture (Supplemental Figure 3, C–F).

Proteomic analyses of heart tissues from animals exposed to oxidative stress as adults or after in utero exposure revealed marked alterations in the cardiac proteome. There were 594 and 441 differentially expressed proteins in the neonates and adults exposed to cardiac H_2_O_2_ compared with controls, respectively (Figure 1K and Supplemental Figure 2, E and F). In both adult and neonatal hearts exposed to H_2_O_2_, highly enriched terms related to energy metabolism, mitochondrial organization, cardiac function, cell death, and oxidative stress. In neonatal hearts exposed to H_2_O_2_, uniquely enriched terms included proteins involved in cardiac development (TNNI1, SGCD, TNNC1), protein translation (SARS, TARS1, EARS2), and mitochondrial biogenesis (TOMM70, OPA1, TIMM13). The adult cardiac proteome was uniquely enriched for terms related to inflammation (PDIA3, STIP1, HSPD1), cardiomyocyte death (ATP2A2, HSPB6, EIF5A), and glucose metabolism (GPI, PGAM2, ENO3), demonstrating both striking similarities and marked differences in the response to oxidative stress between adult and neonatal myocardium (Supplemental Figure 4).

This study reports the development and characterization of a DAAO-TG^Car^ mouse model that permits manipulation of H_2_O_2_ in the heart. We induced cardiac oxidative stress in both adult and neonatal mice and demonstrated substantial impairment in cardiac function, associated with striking changes in cardiomyocyte redox balance and mitochondrial function, along with alterations in the proteome, suggesting a shared pathogenic mechanism. We found marked differences after in vivo oxidative stress across these 2 developmental stages. In vivo cardiac oxidative stress in adult DAAO-TG^Car^ mice recapitulated pathological findings we reported in adult rats (4–6), showing cardiac dysfunction over a period of several weeks in response to d-alanine feeding. In contrast, in utero cardiac oxidative stress markedly affected developing hearts following only days of exposure, underscoring a potential predisposition for cardiac complications later in life. Oxidative stress impairs cardiac regenerative capacity (7), and this model can be utilized to examine effects of in utero versus postnatal oxidative stress on cardiomyocyte maturation. Our neonatal model represents an informative platform to deepen our understanding of the implications of prematurity for the developing myocardium. We propose this chemogenetic approach as a comprehensive model for examining oxidative stress–induced cardiac injury across developmental stages, from in utero to adult exposures. Our model provides insights into the differential effects of oxidative stress on adult and neonatal hearts, establishing a foundation for understanding divergent pathophysiological pathways affected by redox stress across different developmental states.

Further information can be found in Supplemental Methods.

## Supplementary Material

Supplemental data

Supporting data values

## Figures and Tables

**Figure 1:**
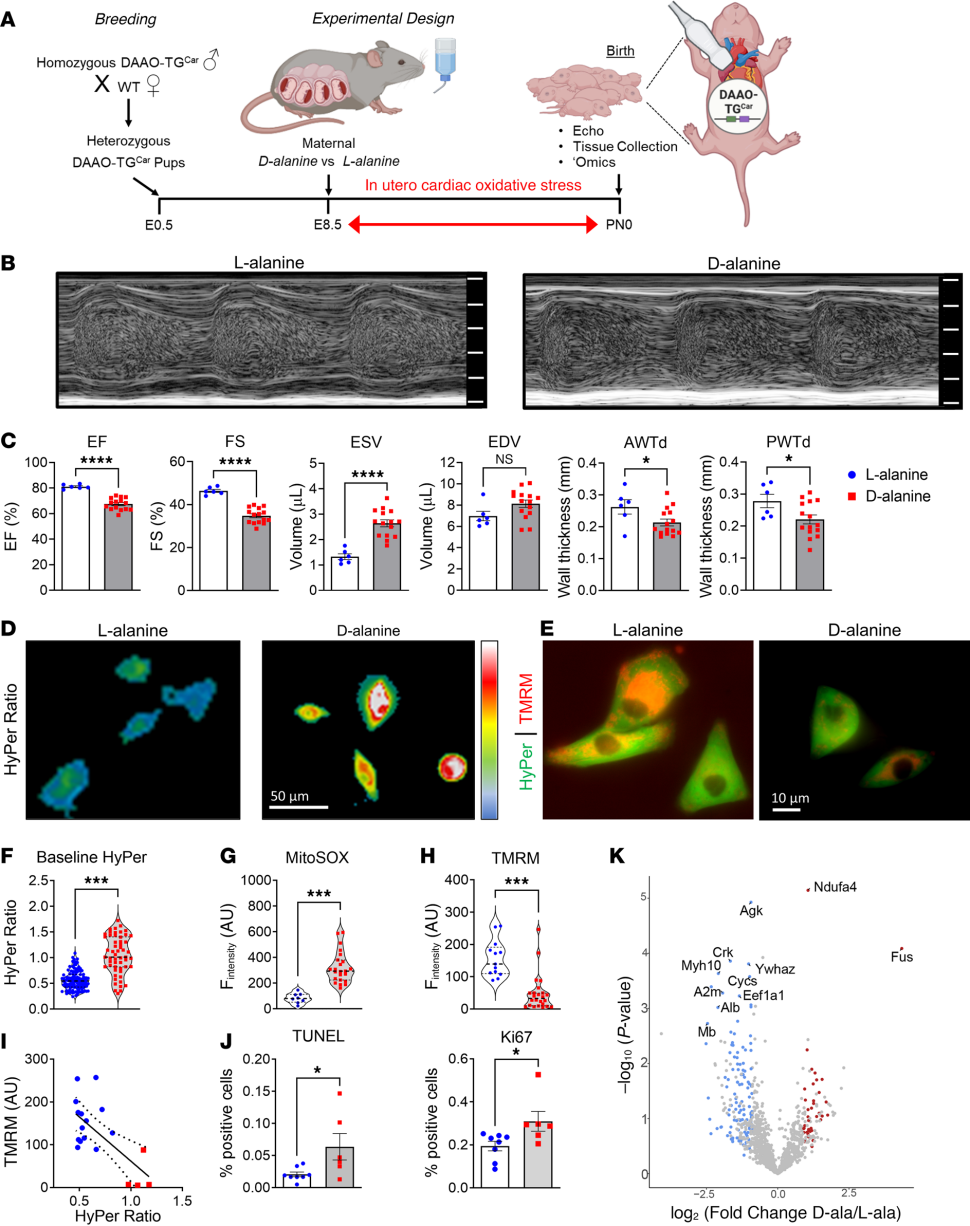
A chemogenetic/transgenic model of neonatal heart failure. (**A**) Breeding strategy for heterozygous DAAO-TG^Car^ pups treated in utero. Created with BioRender.com. E, embryonic day; PN, postnatal day. (**B**) M-mode images of the left ventricle and (**C**) echocardiographic parameters of ejection fraction (EF), fractional shortening (FS), end-systolic (ESV) and end-diastolic (EDV) volume, and anterior (AWTd) and posterior (PWTd) wall thickness in neonates exposed to d-alanine (0.4 M) (red squares) or l-alanine (0.4 M) (blue circles) from E8.5 to PN0. (**D**) Baseline HyPer ratio and (**E**) HyPer and tetramethylrhodamine methyl ester perchlorate (TMRM) images of cardiomyocytes from neonates (PN0) exposed to d-alanine or l-alanine. Quantification of (**F**) HyPer ratio, (**G**) mitoSOX, and (**H**) TMRM fluorescence in arbitrary units (a.U.). (**I**) Correlation between TMRM and HyPer (*r* = 0.857, *P* = 0.0004); dashed lines represent 95% CI. (**J**) Quantification of TUNEL staining (apoptosis) and Ki67 (proliferation). *P < 0.05, ***P < 0.001, ****P < 0.0001 by unpaired *t* test. Values shown as mean ± SEM. (**K**) Volcano plot showing protein abundances in d- versus l-alanine–exposed neonatal hearts; fold-changes and *P* values are log-transformed. Differentially expressed proteins (*P* = 0.05; FDR = 0.1) displayed as upregulated (red) and downregulated (blue). Proteins not significantly changed indicated in gray.

## Version 1 03/14/2024

In-Press Preview

## Version 2 05/01/2024

Electronic publication
