# Supplementary material for: Adult and neonatal models of chemogenetic heart failure caused by oxidative stress
Source: J Clin Invest. 2024 Mar 14;134(9):e178251. doi: 10.1172/JCI178251 (PMC11060721; doi:10.1172/JCI178251)
Supplement: Supplemental data [file jci-134-178251-s227.pdf]

Supplemental Data

Adult and neonatal models of chemogenetic heart failure caused by oxidative stress

Fotios Spyropoulos[1*], Apabrita Ayan Das[2], Markus Waldeck-Weiermair[2], Shambhu Yadav[2], Arvind K. Pandey[2], Ruby Guo[2], Taylor A Covington[2], Venkata Thulabandu[2], Kosmas Kosmas[1], Benjamin Steinhorn[2], Mark Perella[1,3], Xiaoli Liu[1,3], Helen Christou[1], Thomas Michel[2*]

[1]Department of Pediatric Newborn Medicine, Brigham and Women's Hospital, Harvard Medical School, Boston, Boston, USA

[2]Cardiovascular Division, Department of Medicine, Brigham and Women's Hospital, Harvard Medical School, Boston, USA

[3]Pulmonary and Critical Care Division, Department of Medicine, Brigham and Women's Hospital, Harvard Medical School, Boston, USA

*Corresponding Authors:

Fotios Spyropoulos	Thomas Michel
Department of Pediatric Newborn Medicine	Division of Cardiovascular Medicine, Department of Medicine

Brigham and Women's Hospital,
Harvard Medical School,
75 Francis Street Boston,
MA, 02115, USA

Phone: 617-525-8116 (F.S.)

Email: fspyropoulos@bwh.harvard.edu (F.S.), thomas_michel@hms.harvard.edu (T.M.)

Contents:

Supplemental Figures 1-4
Methods
References

Supplemental Figure 1

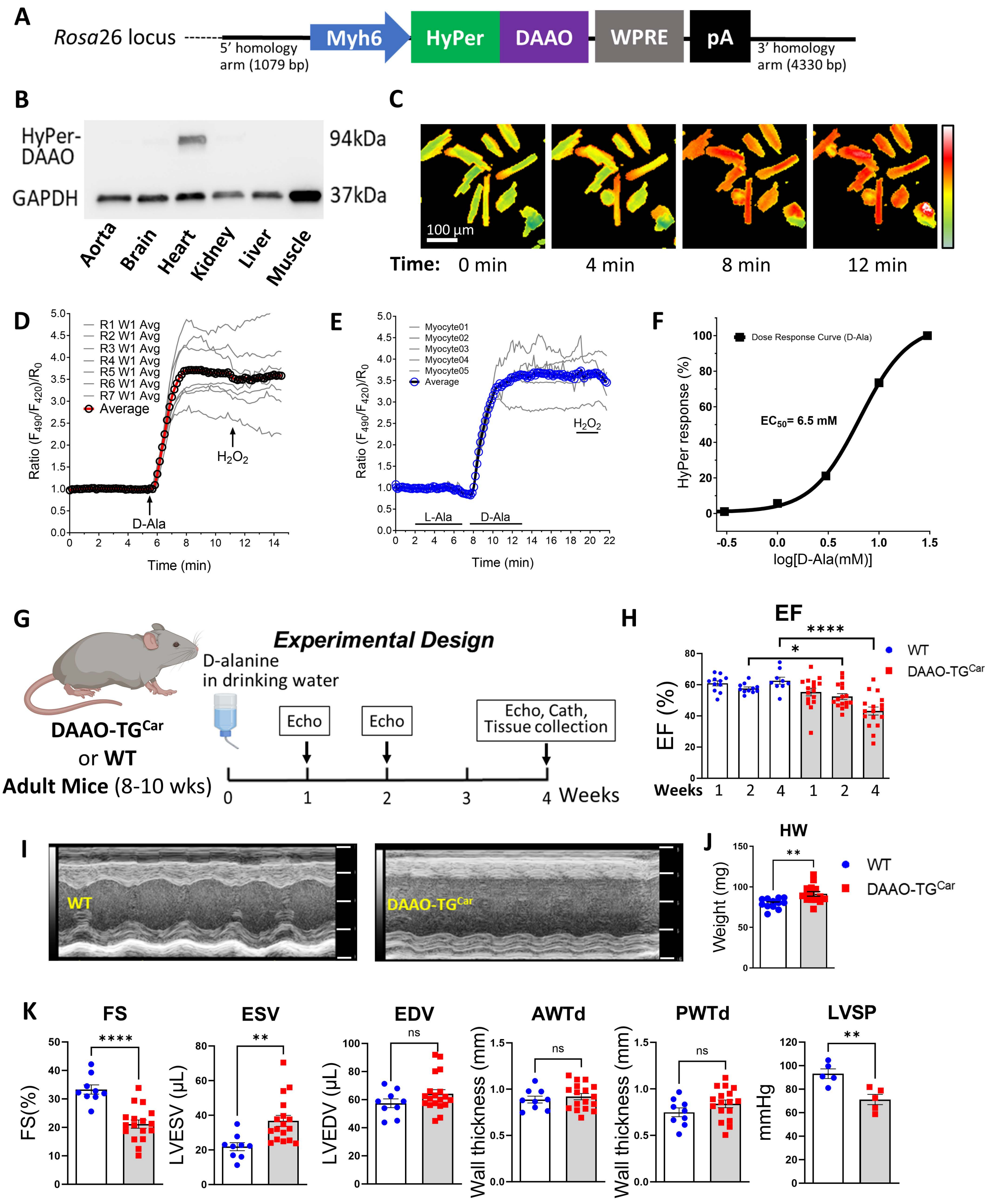

Supplemental Figure 1. A chemogenetic/transgenic model of adult heart failure. (A) Schematic design of the DAAO-TG^{Car} transgenic construct. **(B)** Immunoblot probed with antibodies directed against GFP (GFP recognizes the HyPer component of the DAAO-HyPer fusion protein) in lysates from organ tissues isolated from DAAO-TG^{Car} mice. **(C)** Real-time ratiometric fluorescent images and **(D)** measurements of cardiomyocytes isolated from DAAO-TG^{Car} mice and treated with 20 mM D-alanine (D-Ala) and 25 μM hydrogen peroxide (H_2O_2) for the indicated times. **(E)** HyPer ratio from multiple cardiomyocytes indicating generation of H_2O_2 by DAAO only when treated with 20 mM D-Ala but not L-alanine (L-Ala). **(F)** The HyPer response was analyzed in isolated adult cardiomyocytes across a range of concentrations of D-alanine (D-Ala) starting with 0.5 mM and gradually increasing to 30 mM; at this point, HyPer was maximally oxidized (100% response) with no further increase after addition of extracellular H_2O_2. The EC50 for the HyPer H_2O_2 response was 6.5 mM D-alanine. **(G)** Experimental design of the adult heart failure model. Graphics created with BioRender.com **(H)** Ejection fraction (EF) of DAAO-TG^{Car} and WT mice at different timepoints during D-alanine feeding (0.75 M). Both DAAO-TG^{Car} and WT mice maintain normal cardiac function without provision of D-alanine (Data not shown). However, upon provision of D-alanine to both DAAO-TG^{Car} and WT mice, only the DAAO-TG^{Car} group developed systolic dysfunction as seen by the drop in EF by week 2 of D-alanine feeding and progressed until 4 weeks. We have previously showed that L-alanine feeding did not alter cardiac function in DAAO expressing animals (1). Additionally, in the current study, control animals fed D-alanine have no change in cardiac function. We therefore believe that the observed pathological effects of DAAO reflect the generation of H_2O_2 rather than to non-specific effects of D-alanine absorption and biodistribution. Both D-alanine feeding and DAAO transgene expression are required for the cardiac phenotype. **(I)** Representative M-mode images from short-axis views of the left ventricle in WT and DAAO-TG^{Car} mice after 4 weeks of providing D-alanine

(0.75 M) in the animals' drinking water for both groups. **(J)** Heart weight (HW) and **(K)** echocardiographic parameters of fractional shortening (FS),), end-systolic volume (ESV), end-diastolic volume (EDV), anterior (AWTd) and posterior (PWTd) wall thickness during diastole and LV systolic pressure after 4 weeks of D-alanine (0.75 M) treatment for both DAAO-TG^{Car} (red squares) and WT (blue circles) animals. *$p < 0.05$, **$p < 0.01$, ****$p < 0.0001$ by unpaired Student's t-test. Values are shown as mean ± SEM.

Supplemental Figure 2

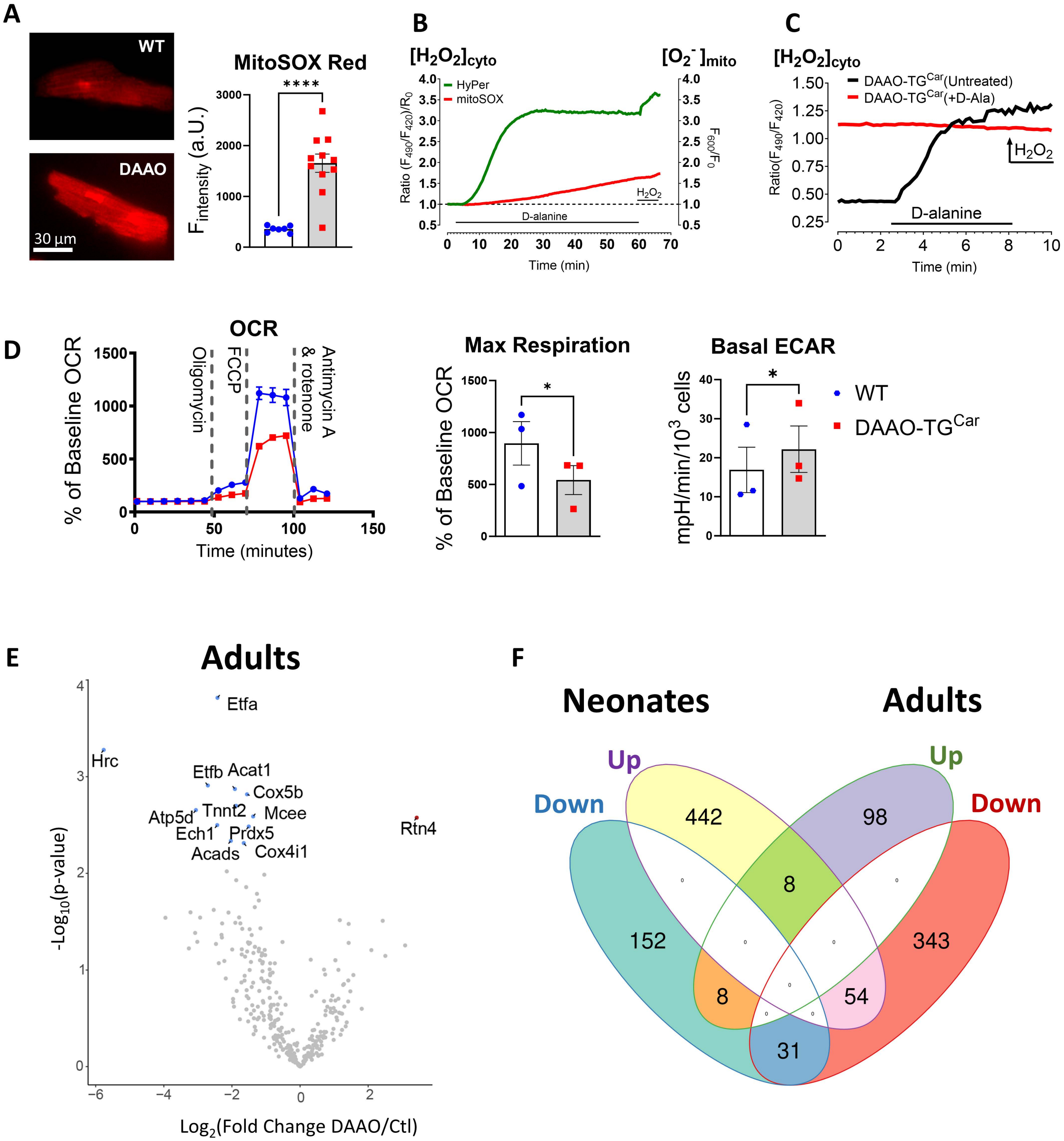

Supplemental Figure 2. Cardiomyocyte redox balance, mitochondrial function and cardiac proteomic analyses in DAAO-TG^{Car} animals. (A) Representative images of cardiomyocytes loaded with mitoSOX, a mitochondrial superoxide ($O_2^{\cdot -}$) indicator, and quantification of mitoSOX fluorescence expressed in arbitrary units (a.U.) from cardiomyocytes which were isolated from adult WT (blue circles) and DAAO-TG^{Car} (red squares) animals after 4 weeks of D-Alanine (0.75 M) feeding. ****$p < 0.0001$ by unpaired Student's t-test. Values are shown as mean ± SEM. **(B)** Representative tracings of co-imaging with HyPer (green tracing) and mitoSOX (red tracing) from cardiomyocytes isolated from DAAO-TG^{Car} mice that were untreated (no *in vivo* D-alanine feeding) and received *in vitro* D-alanine (20 mM) and H_2O_2 (25 μM) after isolation for the indicated times. Addition of D-alanine leads to a rapid increase in cardiomyocyte intracellular H_2O_2 levels followed by a more gradual increase in mitochondrial $O_2^{\cdot -}$ **(C)** Representative tracings of HyPer ratio imaging from cardiomyocytes isolated from DAAO-TG^{Car} mice that were untreated (no *in vivo* D-alanine feeding, black tracing) or after 4 weeks of D-alanine (0.75 M) feeding (red tracing). Myocytes from untreated DAAO-TG^{Car} mice have lower basal oxidation (black line), and the HyPer signal in isolated cardiomyocytes increases in response to addition of D-alanine to the cells. In contrast, myocytes isolated from DAAO-TG^{Car} mice *after exposure to in vivo* D-alanine show a higher basal HyPer signal (red line), which does not increase with the subsequent addition of D-alanine and H_2O_2 *in vitro*, indicating that intracellular redox balance is shifted to a higher level of oxidation as a consequence of *in vivo* D-alanine treatment. **(D)** Oxygen consumption rate (OCR) representative tracing and bar graph of maximum respiration, baseline glycolysis as extracellular acidification rate (ECAR) in cardiomyocytes isolated from WT (blue circles) and DAAO-TG^{Car} (red squares) animals after 4 weeks of D-alanine (0.75 M) feeding. Each data point represents an individual animal (n=3) and the OCR/ECAR rate is determined by averaging the OCR/ECAR rate from 6 technical replicates, with each technical replicate consisting of ~ 2500

cardiomyocytes isolated from either DAAO-TG^{Car} or WT mice. *$p < 0.05$ by paired Student's t-test. Values are shown as mean ± SEM. One limitation of our study is that we did not include untreated animals and did not examine utilization and metabolism of other substrates, including fatty acids, which could also be altered. **(E)** Volcano plot analysis of protein abundances in DAAO-TG^{Car} versus WT adult hearts after 4 weeks of D-alanine (0.75M) feeding. Each dot represents a protein; fold changes and P values are log-transformed. Differentially-expressed proteins (p=0.05; FDR=0.1) displayed as upregulated (red) and downregulated (blue). Proteins not significantly changed indicated in grey. **(F)** Flower plot showing the differentially expressed proteins that are similar and unique in the myocardium of neonates and adults with heart failure.

Supplemental Figure 3

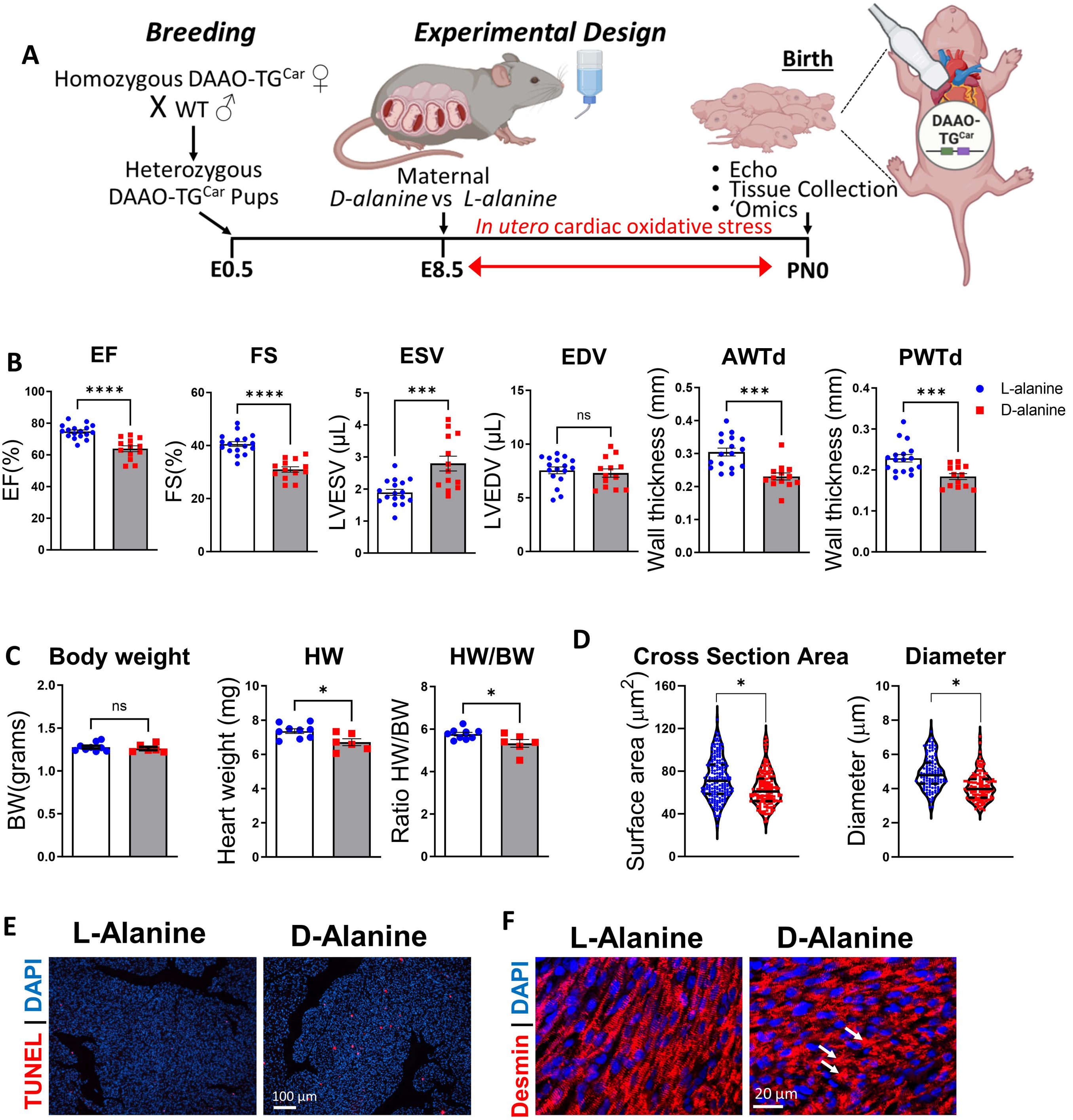

Supplemental Figure 3. Chemogenetic/transgenic model of neonatal heart failure: Alternative breeding strategy and additional histopathologic and echocardiographic characteristics of neonatal DAAO-TG^{Car} heart failure model. (A) Breeding strategy showing schematic to yield heterozygous by breeding WT sires with homozygous transgene-positive DAAO-TG^{Car} dams. Graphics created with BioRender.com. **(B)** Echocardiographic parameters of ejection fraction (EF), fractional shortening (FS), end-systolic volume (ESV), end-diastolic volume (EDV), anterior (AWTd) and posterior (PWTd) wall thickness during diastole in neonates (PN0) exposed to *in utero* D-alanine (0.5M) (red squares) or L-alanine (0.5M) (blue circles) from E8.5 to PN0. *p <0.05, ***p < 0.001, ****p < 0.0001 by unpaired Student's t-test. Values shown as mean ± SEM. E=Embryonic Day; PN=Postnatal Day. **(C)** Body weight (BW), heart weight (HW) and HW to BW ratio from neonates (PN0) exposed to D-alanine or L-alanine. **(D)** Cardiomyocyte cross section area and diameter obtained from fluorescent images of neonatal hearts stained with wheat germ agglutinin (WGA) in red after *in utero* D-alanine (red squares) or L-alanine (blue circles) from E8.5 to PN0. n = 4 animals per group. Data are presented as violin plots; the solid black lines represent median values and dotted black lines indicate quartiles. *p <0.05, by nested t-test. **(E)** Paraffin-embedded sections of neonatal hearts stained with apoptosis marker TUNEL showing increased apoptosis in neonates exposed to *in utero* D-alanine compared to L-alanine controls. Nuclei are visualized after DAPI staining. (Quantification analysis reported in Figure 1). Scale bar, 100 μm. **(F)** Paraffin-embedded sections of neonatal hearts stained with Desmin (Z-disc) specific antibodies showing a disorganized sarcomeric organization with aggregated structures (arrows) in neonates exposed to *in utero* D-alanine compared to those treated with L-alanine from E8.5 to PN0. Nuclei are visualized after DAPI staining. Scale bar, 20 μm.

Supplemental Figure 4

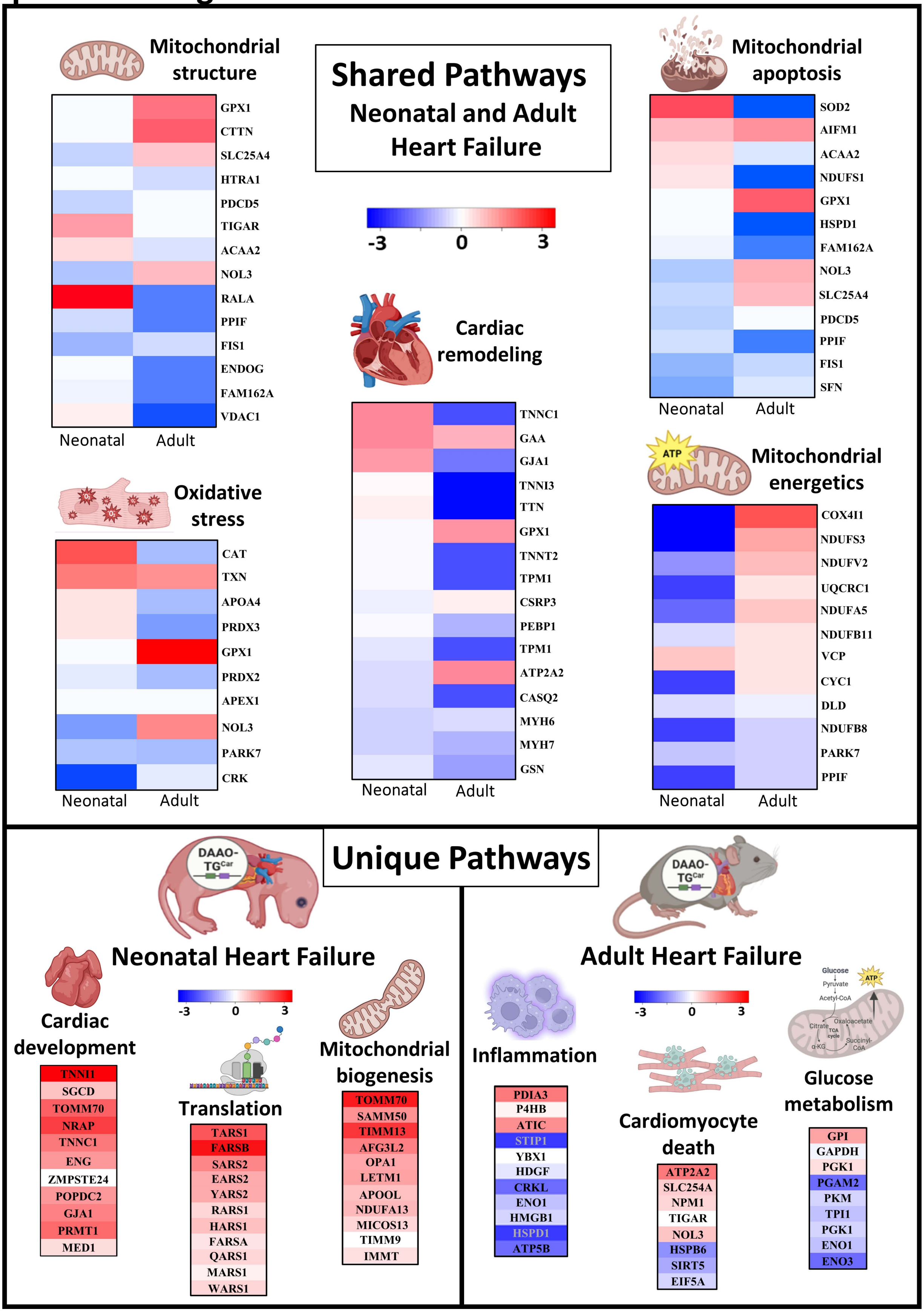

Supplemental Figure 4. Proteome profiling of the heart displays distinct protein signatures in neonatal and adult heart failure. Overview of the shared and unique biological processes based on proteomic analyses of cardiac tissue from neonatal and adult heart failure animals compared to controls. Alterations are defined by upregulated or downregulated proteins with red and blue boxes, respectively, according to log_2 fold change ratio of experimental/control cardiomyocytes. Graphics created with BioRender.com.

Materials and Methods

Sex as a biological variable

Our study examined male and female animals, and similar findings are reported for both sexes.

Mouse Models

Mice were housed (five animals/cage maximum) in cages with unrestricted food (regular diet #5053) and drinking water access in a 12 h light–dark cycle. Room temperature was maintained at 21 ± 2 °C with 35% humidity. Equal numbers of male and female mice were studied, and studies were commenced for the adult mice when the animals were 8-10 weeks of age. A transgenic HyPer-DAAO construct was made in collaboration with Novartis in which the DAAO-HyPer transgene is under control of the cardiac-specific Myh6 promoter to yield the DAAO-TG^{Car} line (Supplemental Figure 1) to target expression to the heart. This construct was directed to the Rosa26 locus by CRISPR/Cas9 methods and transgenic founder lines were generated in C57/Bl6 mice using standard methods; founder lines were identified by PCR, confirming insertion of a single copy of the intact transgenic construct into the Rosa26 locus. All strains are maintained on the C57BL/6 background. All physiological and imaging analyses were performed by personnel blinded to genotype and/or treatment. The homozygous DAAO-TG^{Car} mice develop a similar cardiac phenotype as do the heterozygous DAAO-TG^{Car} mice upon D-alanine provision (data not shown). For this reason, in order to limit the number of experimental animals required and to reduce associated costs, our studies used heterozygous animals. Breeding to generate homozygous mice requires maintenance of larger animal colonies.

Immunoblot analysis

Immunoblotting of tissues was performed as described previously (1); see the Antibodies section below for details.

Live cell imaging

For intracellular H_2O_2 imaging, we followed previously-described protocols (1). In brief, isolated cardiomyocytes from DAAO-TG^{Car} mice were excited at 420 and 490 nm, and emission was captured at 510-530 nm using an Olympus IX81 Microscope equipped with a 20x oil immersion objective (PlanSApo, Olympus) and an ImagEM CCD camera (Hamamatsu, Bridgewater, NJ, USA) at a binning of 4. Data were acquired by MetaFluor Software ((Molecular Devices, San Jose, CA, USA) and intracellular H_2O_2 was quantitated after background subtraction by calculating the ratio of R/R0, where R is the ratio of the 490 nm to the 420 nm signal, and R0 is the baseline 490/420 ratio.

Cultured neonatal or freshly isolated adult cardiomyocytes were either loaded with 3 nM tetramethylrhodamine methyl ester (TMRM, ThermoFisher) or 10 µM MitoSOX™ Red (mitoSOX, ThermoFisher) in Tyrode’s buffer at 37°C for 30 min, and single cell fluorescence was quantitated at 576 nm emission using an Olympus IX81 Microscope. Dual imaging of HyPer Ratio and TMRM or mitoSOX intensity was alternately recorded to correlate levels of cytosolic H_2O_2 with mitochondrial membrane potential or mitochondrial superoxide, respectively. Representative high-resolution images of HyPer or TMRM were processed with MetaMorph Premier Software (vs.7.10.5.476, Molecular Devices) at a binning of 1 using a 40x oil immersion objective (PlanSApo, Olympus) and a digital spinning disc (IX2-DSU, Olympus).

Immunofluorescence and microscopy

In brief, mice were anesthetized using isoflurane followed by intracardiac perfusion with PBS/4% PFA and organ harvest, paraffin embedding, and tissue sectioning. Tissues were processed for immunofluorescence analysis and then incubated with primary antibody overnight at 4° C, washed, and incubated with fluorophore-conjugated secondary antibodies for one hour. For apoptosis, the heart sections were stained with terminal deoxynucleotidyl transferase dUTP nick end labeling (TUNEL), for assessment of cardiomyocyte size, heart sections were stained with wheat germ agglutinin (WGA), isolated cardiomyocytes were stained with phalloidin. Nuclei were visualized with DAPI (4′,6-diamidino-2-phenylindole) staining. Images were captured using a FluoView FV-10i Olympus Laser Point Scanning Confocal Microscope using a 60x objective lens. Confocal filters (Excitation/Emission nm) used for microscopy imaging were: 358/461 (DAPI), 490/525 (Alexa-Fluor488), 594/633 (Alexa-Fluor647).

Antibodies

The following primary antibodies were used: GFP (Cell Signaling Technology catalog number 2956, clone no. D5.1; dilution factor 1:1000); GAPDH (Cell Signaling Technology catalog number 2118, lot. No. 16, clone no. 14C10; dilution factor 1:2000,); DESMIN (Cell Signaling Technology catalog number 5332, clone no. D93F5; dilution factor 1:100)

Isolation of adult cardiomyocytes

Adult mouse cardiac myocytes were isolated from 8–12-wk-old, DAAO-TG^{Car} or WT mice as previously described (2, 3). Adult mouse cardiac myocytes were isolated from 8–12-wk-old C57BL6/J, DAAO-TG^{Car} and WT, mice. The mice were lightly anesthetized with isoflurane, heparinized (50 units, i.p.), and euthanized. The hearts were promptly removed from the chest and retrogradely perfused through the aorta with Ca^{2+}-free perfusion buffer. Subsequently, hearts were

enzymatically digested with type 2 collagenase (Worthington Biochemical, Lakewood, NJ) at a concentration of 2.4 mg/ml in myocyte digestion buffer (2). After 7-8 minutes of digestion, the heart was cut with sterile scissors just below the atria and placed in a 60-mm sterile dish containing 2.5 ml myocyte digestion buffer and the subsequent isolation procedure was performed in a laminar flow culture hood to maintain sterility. The ventricles were separated in small pieces with sterile fine forceps, and then room temperature myocyte stopping buffer was added, consisting of perfusion buffer supplemented with 10% calf serum (HyClone, cat no. SH30073) and 12.5 µM $CaCl_2$. The cardiomyocytes were subsequently mechanically dissociated by gentle agitation. This is followed by the stepwise introduction of Ca^{2+} in myocyte stopping buffer with gradually increasing Ca^{2+} concentrations of 100 µM, 400 µM, 900 µM to a final concentration of 1.2 mM Ca^{2+}. Finally, after Ca^{2+} introduction was complete, the cardiomyocytes were then pelleted, counted, and plated for the respective experiments. In brief, mice were lightly anesthetized with isoflurane, heparinized (50 units, i.p.), and euthanized. The hearts were promptly removed and retrogradely perfused through the aorta with a Ca^{2+} free solution as previously described. Subsequently, hearts were enzymatically digested with type 2 collagenase (Worthington Biochemical, Lakewood, NJ), and cardiomyocytes were mechanically dissociated by gentle agitation. This is followed by the stepwise introduction of Ca^{2+} to a final concentration of 1.2 mM Subsequently, the cardiomyocytes were then pelleted, counted, and plated for the described experiments.

Isolation of neonatal cardiomyocytes

Neonatal cardiomyocytes were isolated upon birth from neonatal mouse hearts. In brief, neonatal mice were rinsed with 75% ethanol solution to sterilize the surface and then decapitated using sterile scissors. The chest was opened along the sternum, the heart was removed and transferred to

sterile dish containing PBS. The heart was washed with PBS, removing other tissues including lungs and atria. The heart was then transferred to a dish containing collagenase I (0.1%) and collagenase II (0.25%) (Worthington Biochemical) in PBS buffer and minced into small pieces using fine scissors. The heart was incubated at 37°Celsius for 50 minutes, and every 15 minutes the tissue was disaggregated using a pipette. Subsequently, digestion was stopped by adding an equal volume of DMEM medium supplemented with fetal bovine serum (FBS, 10%). The cell suspension was filtered through a 70 µm nylon cell strainer, and after centrifugation at 300xg for 5 minutes cells were resuspended in DMEM medium supplemented with 10% FBS and subsequently plated for the described experiments.

Metabolic flux protocol

For extracellular flux measurements, we used a Seahorse Bioscience XF24 Flux Analyzer and cardiomyocytes were seeded in XF Assay Medium (Seahorse Bioscience), supplemented with 1 mM pyruvate, 2 mM glutamine, and 10 mM D-glucose. Preliminary experiments were performed to identify the optimal cell seeding density with 2500 cells per well. The XF24 automated protocol consisted of 10 min delay following microplate insertion, baseline OCR/ECAR measurements [3 x (1.5 minute mix, 2 minute wait, 1.5 minute measure)], followed by injection of port A (56 µl) and OCR/ECAR measurement [3x (1.5 min mix, 2 min wait, 1.5 min measure)], injection of port B (62 µl) and OCR/ECAR measurement [3 x (1.5 min mix, 2 min wait, 1.5 min measure)], injection of port C (69 µl) and OCR/ECAR measurement [3 x (1.5 minute mix, 2 minute wait, 1.5 minute measure)]. Optimal concentrations of oligomycin (2 µM), carbonyl cyanide-p-trifluoromethoxyphenylhydrazone (FCCP, 2 mM), and antimycin A/Rotenone (1 µM each) were diluted in DMSO (Sigma, 154938).

Proteomics: mass spectrometry analysis and data acquisition

We conducted detailed proteomic analyses on heart tissues isolated from animals exposed to oxidative stress as adults or after exposure *in utero* (neonatal hearts). For this analysis, the control group for the adult heterozygous DAAO-TG^{Car} animals fed D-alanine were WT littermates that were also fed D-alanine. For the *in utero* exposure, L-alanine-fed homozygous DAAO-TG^{Car} dams served as controls for the homozygous DAAO-TG^{Car} dams fed D-alanine. One of the limitations of this study is that we did not include animals that were untreated as a control group. However, control WT animals that were fed D-alanine showed no change in cardiac function whatsoever. We can therefore conclude that the observed pathological effects of the DAAO-TG^{Car} group is due to the generation of H_2O_2 by DAAO.

Hearts from adult and neonates were removed after sacrificing the mice. Tissues were washed in ice cold PBS. Protein was then isolated in RIPA buffer after mincing and homogenizing the tissue in the same buffer. Total protein was then precipitated with TCA and resolubilized in Rapigest SF (Waters, Milford, USA). Resolubilized proteins were processed for further mass spec analysis as previously described (4). 2 ml of modified sequencing-grade trypsin (20 ng/ml) (Promega, Madison, WI) was added into each sample and the samples were placed in a 37ºC bath overnight. Samples were acidified by spiking in 20ml of a 20% formic acid solution and then desalted using a STAGE tip (5).

On the day of analysis, the samples were reconstituted in 10 µl of HPLC solvent A. A nano-scale reverse-phase HPLC capillary column was created by packing 2.6 µm C18 spherical silica beads into a fused silica capillary (100 µm inner diameter x ~30 cm length) with a flame-drawn tip (6). After equilibrating the column each sample was loaded via a Famos auto sampler (LC Packings,

San Francisco CA) onto the column. A gradient was formed, and peptides were eluted with increasing concentrations of solvent B (97.5% acetonitrile, 0.1% formic acid). As peptides eluted, they were subjected to electrospray ionization and then passed through an LTQ Orbitrap Velos Elite ion-trap mass spectrometer (Thermo Fisher Scientific, Waltham, MA). Peptides were detected, isolated, and fragmented to produce a tandem mass spectrum of specific fragment ions for each peptide. Peptide sequences (and hence protein identity) were determined by matching protein databases with the acquired fragmentation pattern by the software program, Sequest (Thermo Fisher Scientific, Waltham, MA) (7). All databases include a reversed version of all the sequences and the data was filtered to between a one and two percent peptide false discovery rate. We used 1 unique peptide as a threshold to identify the proteins and the quantification was performed based upon the intensity. The number of annotated proteins included a total of 632 proteins that were identified in the adult cardiac proteome, and 698 proteins were identified in the neonatal cardiac proteome. We identified 127 common proteins between the adult and neonatal cardiac proteome. The label free quantification values of the annotated proteins were normalized by $\log_2$ transformation. Pairwise comparison between group was done by t test and one way ANOVA using R package Limma (version 3.50.0). Differences according to Benjamin- Hochberg adjusted p value <0.05 was considered significant. PCA analyses of the proteins were carried out by R package application (version 5.4.1). Volcano plots were constructed using R with FDR value of 0.1. The online tool DAVID was used to annotate biological processes via GO analysis. Phenotypic analyses were performed with MGI database. Heatmaps were created using R package ComplexHeatmap (version 2.10.0). Venn diagram was built using the R package VennDiagram.

Echocardiography

Echocardiography was performed as previously described (1) using a Visual Sonics 2100 system equipped with a MS-550 probe for adults and MS-700 probe for neonatal mice. The adult mice were initially anesthetized with 2-3% isofluorane and then titrated to 1-1.5% isofluorane to maintain heart rate above 450 bpm. The neonatal mice underwent echocardiography with gentle restraint and without sedation. Standard echocardiographic images of mid-papillary level short-axis and long-axis views of the heart were obtained. M-mode images were analyzed with Vevo LAB software (V.3.1.1 FUJIFILM Visualsonics, Toronto, Canada). The sonographer and analyzer were blinded to the experimental treatment and/or genotype.

Statistical analyses

Statistical analysis for in-between group comparisons was performed using Student's t-test (for two group comparisons). Spearman correlation was performed using GraphPad. Data values are presented as individual data points and expressed as means ± standard error of mean (SEM). Individual statistical tests are described in the corresponding figure legends. A p value of < 0.05 was considered statistically significant. Equal numbers of male and female mice were studied. All physiological, and imaging studies were performed and analyzed by scientists blinded to genotype and treatment. Statistical analyses were performed using GraphPad Prism 11.0 (GraphPad Software, La Jolla, CA).

Study approval:

All animal experiments were carried out under NIH guidelines for the care of laboratory mice, and all animal protocols were approved by the Brigham and Women's Hospital Institutional Animal Care and Use Committee (protocol 2016N000278).

Graphics:

Graphics including icons, templates and other original artwork appearing in the figures of this manuscript have been created with BioRender.com, agreement number PR26IQJ766.

Data and materials availability:

Data supporting the findings of this study are available in the article and its Supplementary Information. The mass spectrometry proteomics data have been deposited to the ProteomeXchange Consortium via the PRIDE (8) partner repository with an accession ID (PXD049254). Source data are provided with this paper and are available in the supporting data values.

Author contributions:

Conceptualization: FS, TM

Methodology: FS, BS, MP, HC, TM

Investigation: FS, AD, MWW, XL, KK, RG, TC, VT, SY, AP

Supervision: FS, TM, MP, HC

Writing – original draft: FS, TM

Writing – review & editing: FS, TM, AD, MWW, XL, KK, RG, TC, VT, SY, HC, AP

Acknowledgments:

This study was supported by National Institutes of Health (NIH) grants T32 HL007609 and K08 HL168240 to F.S.; NIH grants R33 HL157918, R21 AG063073 and R01 HL152173 to T.M.; FWF

fellowship award J4466-B to M.W.W. We would like to acknowledge Novartis Institutes of Biomedical Research (NIBR) for provided support for development of the DAAO-TG^{Car} mouse founder lines, and William Chutkow (NIBR) provided important guidance in transgenic line design and identification. We acknowledge the BWH Seahorse core and William Oldham, M.D., Ph.D. for his guidance on the extracellular flux measurements. We acknowledge Bonna Ith for his assistance with colony management, timed pregnancies, as well as histology services.

References

1. Steinhorn B, et al. Chemogenetic generation of hydrogen peroxide in the heart induces severe cardiac dysfunction. *Nat Commun.* 2018;9(1):4044.
2. O'Connell TD, et al. Isolation and culture of adult mouse cardiac myocytes. *Methods Mol Biol.* 2007;357:271-96.
3. Sartoretto JL, et al. In vivo imaging of nitric oxide and hydrogen peroxide in cardiac myocytes. *Methods Enzymol.* 2013;528:61-78.
4. Yadav S, et al. Sensory ataxia and cardiac hypertrophy caused by neurovascular oxidative stress in chemogenetic transgenic mouse lines. *Nat Commun.* 2023;14(1):3094.
5. Rappsilber J, et al. Stop and go extraction tips for matrix-assisted laser desorption/ionization, nanoelectrospray, and LC/MS sample pretreatment in proteomics. *Anal Chem.* 2003;75(3):663-70.
6. Peng J, and Gygi SP. Proteomics: the move to mixtures. *J Mass Spectrom.* 2001;36(10):1083-91.
7. Eng JK, et al. An approach to correlate tandem mass spectral data of peptides with amino acid sequences in a protein database. *J Am Soc Mass Spectrom.* 1994;5(11):976-89.
8. Perez-Riverol Y, et al. The PRIDE database resources in 2022: a hub for mass spectrometry-based proteomics evidences. *Nucleic Acids Res.* 2022;50(D1):D543-D52.
